# Neuromarkers for Mental Disorders: Harnessing Population Neuroscience

**DOI:** 10.3389/fpsyt.2018.00242

**Published:** 2018-06-06

**Authors:** Lee Jollans, Robert Whelan

**Affiliations:** School of Psychology and Trinity College Institute of Neuroscience, Trinity College Dublin, Dublin, Ireland

**Keywords:** biomarker, neuromarker, machine learning, nosology, population neuroscience, big data, research domain criteria, neuroimaging

## Abstract

Despite abundant research into the neurobiology of mental disorders, to date neurobiological insights have had very little impact on psychiatric diagnosis or treatment. In this review, we contend that the search for neuroimaging biomarkers—neuromarkers—of mental disorders is a highly promising avenue toward improved psychiatric healthcare. However, many of the traditional tools used for psychiatric neuroimaging are inadequate for the identification of neuromarkers. Specifically, we highlight the need for larger samples and for multivariate analysis. Approaches such as machine learning are likely to be beneficial for interrogating high-dimensional neuroimaging data. We suggest that broad, population-based study designs will be important for developing neuromarkers of mental disorders, and will facilitate a move away from a phenomenological definition of mental disorder categories and toward psychiatric nosology based on biological evidence. We provide an outline of how the development of neuromarkers should occur, emphasizing the need for tests of external and construct validity, and for collaborative research efforts. Finally, we highlight some concerns regarding the development, and use of, neuromarkers in psychiatric healthcare.

## Introduction

According to figures from the World Health Organization, the projected risk for developing some form of mental disorder across the lifetime is between 18 and 55% (1). Globally, mental disorders are the leading cause of years lived with disability (2). Thirty-eight percent of the EU population is estimated to suffer from a mental disorder each year (3). In 2010, the estimated average cost of addictive, anxiety, mood, and psychotic disorders was more than €3,500 per affected individual in Europe (4). The corresponding figure for dementia was as high as €16,500 per individual. The staggering economic cost and disability burden of mental disorders indicate that research into improved prevention and treatment is imperative. Unlike most other areas of medicine, prolific research in psychiatry throughout the past half century has not led to any substantial changes in treatment approaches or in the conceptualization of diagnostic categories. The last major shift in how mental disorders are treated occurred in the 1960's with the introduction of psychoactive medications, following the growing recognition that mental disorders have a basis in biology. However, the neuropathological underpinnings of psychiatric conditions have little influence on current healthcare practice, with both diagnosis and prognosis relying primarily on observed symptoms or self-report.

In this review we will explore this gap between neuropsychiatric research and psychiatric healthcare. First we will provide a general overview of how neuropsychiatric research is conducted, in order to familiarize the reader with the concepts referenced in later parts of the text. Second, we will detail the criteria that must be fulfilled for neuropsychiatric research to be clinically useful, introducing the concept of neuroimaging biomarkers. Third, we will highlight the key issues that have impeded the application of insights gained from neuropsychiatric research into applied psychiatric settings. Fourth, we will address these issues and offer solutions. Fifth, we will discuss some of the considerations which researchers and clinicians should take into account when carrying out research with, or when using biological models of mental disorders. Finally, we will provide a summary and a set of recommendations for neuropsychiatric research, to make it more clinically useful. A glossary explaining some of the terms used in this review is provided.

## The brain and mental disorders—an overview of neuropsychiatric research methods

From the fallacious discipline of phrenology to modern neuroimaging, researchers have hoped that understanding the brain would provide explanations or justifications for behavior, personality traits, cognition, and affect. The earliest knowledge of the connection between brain and behavior comes from post mortem examinations and studies of patients with brain lesions. A famous example is the case of Phineas Gage, whose personality changed dramatically after an iron rod passed through his skull and destroyed much of his frontal lobe. Cases of dramatic changes in patients who experienced brain lesions were the first evidence that some brain functions rely on specific brain areas. With the advent of non-invasive imaging technology, neuroscientists have no longer had to rely on lesion studies to explore the neurophysiological basis of cognitive functions, behavior, and pathology. Magnetic Resonance Imaging (MRI) is a non-invasive imaging technology which provides clinically useful images of internal tissue and organs. MRI scanners have been used since the 1980s and are available in almost all hospitals in the developed world. MRI can be used to examine brain structure and to measure gray and white matter volume in the brain. Functional MRI (fMRI) has been used for brain imaging since the early 1990's, and has provided many valuable insights into psychopathology, cognition, and behavior (5–7). fMRI utilizes regional blood-flow in the brain to infer neuronal activity via the blood-oxygen-level dependent (BOLD) signal. Most fMRI studies manipulate some variable of interest, such as the visual or auditory stimuli individuals are exposed to, and examine the difference in BOLD signal specifically associated with that variable. These studies can reveal how activations in specific brain regions are associated with certain types of sensory or cognitive processing.

There is a rich neuroimaging literature examining psychiatric pathology. Psychiatric neuroimaging research typically involves a group of patients, and a group of healthy control participants (normally matched to the patient group in terms of various demographic characteristics). These are compared in terms of their brain structure or function. The typical sample size of a neuroimaging study from a single laboratory does not exceed 100 participants. In contrast, neuroimaging datasets typically include hundreds—if not thousands—of voxels (see Glossary) or regions of interest (ROIs, see Glossary), particularly when data from multiple modalities are used (such as MRI and electrophysiological recordings or positron emission tomography). MRI and fMRI data are usually analyzed by carrying out statistical significance tests on each voxel. This type of analysis is referred to as mass-univariate analysis, as it involves conducting a massive amount of tests for each analysis. When groups of patients and control participants are being compared, an ANOVA or *t*-test (see Glossary: Inferential Statistics) will usually be carried out at each voxel. To account for the high risk of false positive findings (see Glossary), mass univariate analyses are ordinarily reported using corrected statistical significance thresholds. This approach has produced important insights into the neuropathology underlying many psychiatric conditions including addiction [e.g., (8)]; schizophrenia [e.g., (9)]; social anxiety disorder [e.g., (10)], Attention deficit hyperactivity disorder [ADHD; e.g., (11)], and anorexia nervosa [e.g., (12)]. However, there are considerable issues in terms of reliability, generalizability, and reproducibility with this type of analysis framework in terms of identifying neuromarkers (see Glossary). We outline the problematic elements of this approach in the section Barriers to the Use of Neuromarkers in Applied Psychiatry.

### Summary

In this section we provided a brief outline of how neuropsychiatric research investigating mental disorders is often carried out. In the past two decades MRI has become the main tool used to investigate brain structure and function. Mental disorders are usually studied by comparing a group of individuals diagnosed with the mental disorder to a group of healthy control participants. Groups are then compared using mass-univariate analyses to investigate possible group differences.

## Biological models in psychiatry—what they should look like

In this section we will first outline why biological models (see Glossary) would be beneficial in psychiatry, introducing the concept of biomarkers. Subsequently we will describe some of the key characteristics which a useful biomarker must have.

### Biomarkers and why psychiatry needs them

As previously noted, diagnoses of mental disorders are based on observed and/or self-reported symptoms, which are highly heterogeneous within, and often common across disorders (13). The absence of clear and distinct disorder phenotypes and a high rate of comorbidity of psychiatric disorders pose a considerable challenge to clinicians when it comes to selecting a treatment pathway from which the patient is most likely to benefit. In other domains of medicine, predictive models for estimation of treatment efficacy, risk assessment, and prognosis are routinely employed by medical professionals, and advocated by policymakers (14). Over the last decade, for example, cancer and heart disease are two specific areas in which biologically based (predictive) models, or *biomarkers*, have been used for purposes of screening, diagnosis, staging, prognosis, treatment selection, and monitoring (15–17). Rather than replace the clinician, these biomarkers provide a measure that can supplement clinical decision-making (18, 19). This affords patients and healthcare providers the opportunity to implement preventative measures in high-risk patients, to identify a disease in its early stages, aid differential diagnosis, select treatment pathways that are most likely to benefit the patient, and to make a well-informed prognosis about treatment outcome and disease course. Being able to estimate the likelihood that a patient will respond to a particular treatment is the basis for precision medicine, and for the integration of diagnosis and therapeutics [“theranostics,” (20)]. Based on predicted treatment response or disease course, clinicians can personalize treatment plans and avoid or delay costly, arduous, and possibly ineffective treatments. This would have a great impact on the quality of life of patients, and on the economic and personal cost of healthcare to the individual and society.

In order to be clinically useful, a biomarker needs to augment existing diagnostic/prognostic criteria. That is to say, the estimate of a future event (or current condition) based on the biomarker, or adding the biomarker to current methods, needs to be better than the estimate based on current methodology alone. A key element of why biomarkers are so desirable in medicine is that they provide an objective estimate. This has the potential to reduce bias in clinical decision making. In psychiatry, the incorporation of biological evidence into diagnosis, prognosis, and treatment selection could improve the quality of healthcare which patients receive (21). The National Institute of Mental Health acknowledged this in their “Research Domain Criteria” (RDoC; www.nimh.nih.gov/research-priorities/rdoc/index.shtml) almost a decade ago. The RDoC framework assumes that (1) mental disorders are disorders of brain circuits, (2) neuroscientific methods can identify these dysfunctions *in vivo*, and (3) genetic and imaging data will yield biomarkers that can augment clinical management (22).

### What a biomarker should look like

In practical terms, a good biomarker needs to be workable—it must be reasonably simple and quick to obtain the data necessary to compute the biomarker, so that clinicians can realistically implement the measures in assessments (13). It is easiest to implement unimodal models (see Glossary) in new settings, as they do not require multiple imaging protocols or modalities. A measure that is easy and practical to include in an assessment protocol should also be low in personal and economic cost. Paying for an MRI scan for the sake of a small improvement in diagnostic accuracy may not be worthwhile. Yet, as Gabrieli et al. (21) point out, a neuromarker may provide sufficient improvement in diagnostic or prognostic accuracy to be a cost-effective option. If the human and economic cost associated with delaying treatment or administering a treatment that is ineffective can be prevented or reduced, then administering an MRI may be more economical than the alternative. However, Gabrieli et al. (21) also note that to be clinically useful the question that must be answered is not solely whether one particular treatment is likely to work, but which treatment out of a number of treatment options is likely to be the most beneficial for the patient. Another practical concern is that the imaging protocol necessary for calculation of the neuromarker must be robust to slight deviations in data collection or preprocessing procedure. That is to say, broadly similar results should be obtained when different clinicians or professional health-care providers administer the test, or when different participants view similar stimuli thought to engage the same sensory or cognitive processes (23). Furthermore, a good biomarker must have good construct validity. A classifier which purports to identify individuals with Alzheimer's disease should also perform reasonably well identifying individuals with mild cognitive impairment, but should have no relevance when separating unipolar from bipolar depression.

#### Summary

In this section we have contended that the integration of neuromarkers into the diagnosis and treatment of mental disorders would be of great benefit to both patients and clinicians. Considering the high economic and personal cost of mental disorders, neuroimaging biomarkers may prove to be valuable and cost-effective. Useful biomarkers must be easy to implement in new settings, and have good external validity.

## Barriers to the use of neuromarkers in applied psychiatry

Reasons and possible solutions for the discrepancy between neuroscientific research and clinical applicability have been discussed by many researchers and clinicians (20–30). Four areas are consistently identified as targets for improvement in translational neuroscientific research: (1) the statistical approaches used in neuroscience, (2) the need for larger population-based samples, (3) a lack of mechanistic understanding of psychiatric neuropathology, and (4) the need for a move away from the often ill-defined phenomenological (see Glossary) diagnostic criteria in psychiatry. In this section we will address each of these areas, outlining why they pose a threat to the clinical applicability of neuroimaging research. Solutions to these issues will be put forward in the subsequent section.

### Statistics and study design

Most neuroimaging studies use group differences to infer characteristics of neuropathology. While the knowledge of how brain structure and function differs between patient populations and control subjects is valuable in terms of understanding disease mechanisms, making inferences about cognitive or affective processes based on observations of brain structure or function (i.e., reverse inference) is problematic (31). However, a key reason for the inability of neuroscientific insights to translate into clinical practice is the reliance on the results of inferential statistics (see Glossary) to determine the relevance of results, which does not necessarily translate into clinical relevance. In an applied setting only those variables that can generate some information about the outcome of interest for an individual patient—whether this is the projected disease course or simply whether or not a patient fits into a specific diagnostic category—are useful. Statistical significance between groups is quantified based on group means and within-group variance [see (32) for a discussion]. Differences will therefore be strongest between groups with high within-group homogeneity. Good predictors, on the other hand, capitalize on heterogeneity within the entire sample to generate an outcome estimate. While variables that significantly differ between groups may also be good predictors, this is not necessarily the case, and vice versa (20, 23, 32, 33).

Another concern regarding current statistical standards in neuroscience is the reliance on mass-univariate analyses to determine statistical significance of results. Considering each voxel in isolation assumes a level of extreme localized functional specialization that does not reflect the network-based neurophysiological underpinnings of cognitive functions and clinically relevant outcomes (34). The inherent connectedness of neuroimaging data necessitates that determining the predictors of a cognitive, behavioral, or clinical outcome should examine any interaction effects between brain regions. Examining a single cluster of voxels or a single brain region is rarely very informative. Multivariate, as opposed to univariate analysis procedures encompass the simultaneous analysis of more than one independent variable. In the context of neuroimaging research this typically takes the shape of multivariate (or multi-voxel) pattern analysis or regression analyses embedded within a machine learning approach. Most of these can be grouped into (1) classifiers or logistic regression approaches, and (2) linear regression approaches. Multivariate statistical tools have been incorporated into neuroimaging research more and more in the past decade (34). However, there is an additional concern when using multivariate methods. Neuroimaging data are expensive to acquire (approximately €750 per hour) and consequently sample sizes in neuroimaging research are generally quite small. The number of input variables in a neuroimaging datasets often exceeds the number of observations (i.e., sample size). When this is the case, predictions are at a high risk of being overly optimistic (35). This occurs when a model fits to the idiosyncrasies of the sample rather than factors that are common to the population from which the sample is drawn. This is generally referred to as “overfitting” [see (36) for an overview of this issue in neuroimaging]. Overfitting leads to models producing very good predictions on the sample they were created with, but that then generalize poorly to other samples from the same population.

### Understanding mechanisms

Besides improving the accuracy of clinical judgements, biological models of psychiatric illness have the potential to illuminate the neurobiological mechanisms of disease etiology and recovery. However, at least some understanding of neuropathology is necessary to create good neuromarkers (34). In the psychological tradition, most mental disorders are associated with at least one theoretical model of the component processes and functions associated with maladaptive behavior, cognition, or affect. Such models can be applied to neuroimaging data to examine associations between brain structure or function and theoretical components of the model [e.g., (37)]. This approach has the potential to illuminate certain aspects of neurobiology in the light of the psychological model, but rests on the assumption that the model reflects a cognitive process accurately (38). The majority of theories about maladaptive behavior or psychiatric pathology combine neuroscientific evidence with assumptions about the psychological processes that they influence or support. Theoretical models can emerge from neuroscientific evidence, or neuroscientific evidence can lead to the confirmation or reconsideration of already established psychological models. An example of neuroscientific evidence leading to a reconsideration of a theoretical model comes from the field of addiction, where increased availability of neuroimaging research led to an alteration in how the role of reward processing was viewed (39). This example shows that the state of understanding disorder mechanisms depends both on the available neuroscientific evidence, and on the available theoretical models of a condition.

Another issue regarding the understanding of disorder mechanisms emerges with the use of multivariate statistics. While the variables (“features”) which are included in the model may be determined by the current understanding of disorder mechanisms, the model building process itself will be divorced from the theoretical understanding of the condition. This makes it important that neuromarker models be interpretable. That is to say, it must be possible to determine whether a neuromarker model is neurophysiologically plausible (34). As Woo et al. (34) put it, it is difficult to know when and why a model will fail if it is not understood why it works in the first place. However, constructing good interpretable models from neuroimaging data is not an easy feat. The machine learning tools which have been adopted by neuroimaging researchers typically come from fields such as computer science or engineering, where the importance that is placed on model interpretability is much lower.

### Psychiatric nosology

Nosology in psychiatry does not have a biological basis, and incorporates no knowledge about neuropathology. Evidence-based efforts to redefine diagnostic categories have been made using cluster analysis (see Glossary). Leaning on the psychological history of clustering psychiatric populations based on neurocognitive and affective symptoms, a phenomenological subclassification of a population can be achieved. This can then be linked to neuroimaging data to reveal possible biological subtypes of the disorder. An example where neuromarkers could be useful is the case of bipolar disorder and schizophrenia. These disorders are distinct in terms of their diagnoses (40) but may in fact have a shared etiological pathway (41, 42). Both these disorders pose a considerable challenge in terms of differential diagnosis, as they have substantial commonality in their symptomatology (see Glossary). There is considerable evidence that biological subtypes of mental disorders may not necessarily correspond to diagnostic categories [see also (43, 44) for a discussion of biological subtypes in ADHD]. However, this knowledge alone has limited clinical applicability because no relationship to disorder etiology, treatment outcome, or disorder trajectory was established. Nevertheless, it is crucial to consider that neuromarkers may be unattainable when working with disorders based on phenomenology and current diagnostic categories [RDoC; (45)]. Therefore, the goal of the RDoC is a diagnostic system in psychiatry which is based on an understanding of the biological and psychosocial basis of mental illness (45). Many researchers have asserted that the unreliable (46) symptom-based stratification of mental disorders has prevented progress in determining the etiology and pathophysiology of mental disorders (20), and that nosology should be recast in more biologically meaningful terms, based on neuroscientific evidence (20, 47). An important consideration is that any categorization of disorder subtypes based on biological data will likely impose artificial boundaries upon the spectrum of disease pathology and symptomatology. Therefore, a biological redefinition of disorders must reflect the concept that biological indicators of pathology are likely to exist on a continuum.

#### Summary

In this section we have outlined the central problems which have led to neuroimaging research largely failing to generalize to clinical psychiatric practice. A fundamental issue in neuroimaging research is the mass-univariate analysis framework often used. When moving to multivariate approaches it is important to remain mindful of the danger of overfitting. Overfitting is more likely to occur when sample sizes are too small or when the number of variables is too large. Furthermore, neuroimaging models should be interpretable, to ensure their neurophysiological plausibility. Lastly, it is problematic to rely on current diagnostic categories when creating neuroimaging models of mental disorders, as these categories are often ill defined.

## Neuromarkers—a recipe

In this section we will address the challenges raised in the previous section. We will address each point by describing methods which are already being used in the field to improve neuromarker research. This section will be structured to follow the lifecycle of neuromarker development, focusing on the following elements: Study design, analysis frameworks, statistical tools, the extended development pipeline of a neuromarker, and an example of a neuromarker which has already progressed through much of this developmental pipeline.

### Study approaches

Dubois and Adolphs (23) likened big data in neuroscience to accelerators in particle physics or telescopes in astronomy—a necessary tool for scientific progress [for a discussion of the role of big data in psychiatry see also (30)]. Large samples are achievable through multi-site imaging initiatives and consortia like the Alzheimer's Disease Neuroimaging Initiative [ADNI, (48)], IMAGEN (49), EU-AIMS (50, 51), the Adolescent Brain Cognitive Development Study (NIH), the Human Connectome project (52), and ENIGMA (53). However, not all data from these initiatives are publicly available. Another option to achieve large sample sizes is data-sharing, possible through data-sharing facilities such as NeuroVault [neurovault.org, (54)] and OpenfMRI [openfmri.org, (55)]. While utilizing data from multiple laboratories and multiple geographic locations contributes to the validity and generalizability of models, collapsing data across multiple data collection sites is a non-trivial task that can introduce additional confounding factors (24). While complex, it is possible to combine data from multiple data collection sites into a well-performing model (56). Large datasets facilitate a population-based approach to neuromarker research. Large studies like IMAGEN not only gather neuroimaging data, but also gather information on genetics, demographics, and life history. This makes it possible to examine psychopathology in a holistic manner (57), under the rubric of “population neuroscience.” By taking into consideration information from other domains, neuromarkers can more meaningfully contribute to our understanding of the etiology of psychopathology.

Many large datasets include participants with a wide range of symptoms. Yet, studies using these data to identify neural signatures associated with mental disorders often select a fairly narrow subset of cases and matched controls. Studies using well-defined and clearly distinct groups are able to classify between patients and controls or between patient groups. However, the context in which knowledge gained from such classification studies is useful must be carefully considered at the outset of designing such a study. For example, a classifier which can differentiate between patients with schizophrenia and patients with bipolar depression with high accuracy only has clinical utility when it is applied to a patient who is already known to fall into either of those groups. If however the individual were suffering from unipolar depression the outcome of the classifier would have little meaning. Although strictly controlling for variables such as age, socio-demographic circumstances, symptoms, or medication use gives the experimenter greater control and greater clarity over the source of an effect, restrictions on study inclusion also restrict the utility of findings. That is to say, stricter inclusion criteria also narrow the range of circumstances in which a model will be useful and applicable (34). Considering this restriction on how a model can be useful in practice, the models and neuromarkers that will have the highest clinical significance will be models that take into account the heterogeneity within the population, and ideally define pathology in a continuous rather than binary fashion (19, 20, 34). This is particularly important when attempting to predict clinical outcomes such as future psychopathology. Large datasets make it possible to create neuromarkers that provide information about how an individual's brain activity differs from the population average. This provides insight into how linear variations in brain structure and function are associated with changes in a variable of interest on a spectrum which includes the population-mean and pathological manifestations. In comparison to case-control studies, this individual-difference approach would mark a move toward creating neuromarkers for certain symptom clusters or processing domains, rather than for specific diagnoses.

Attempting to identify neural signatures of individual types of processing or behavior can be seen as a “component process” approach [(34), p. 371]. This would ideally result in a set of models which capture brain structure or function associated with a particular variable that linearly varies across the population. A number of such models could then be combined to identify specific populations. This approach would be very valuable in terms of risk assessment, such as early identification of adolescents at risk for future psychopathology. An example of this could be ADHD and substance use disorder. Both individuals with ADHD, and individuals with substance use disorder often show poor inhibitory control. A neuromarker that measures inhibitory control should therefore provide similar estimates for these two groups. Identifying an adolescent's level of inhibitory control based on a neuromarker can therefore provide a measure of risk of maladaptive behaviors involving poor inhibitory control. The component process approach is thus very well-suited to addressing certain types of research questions, such as general risk for maladaptive behavior. Applying the component process approach to other questions, such as predicting response to treatment, may however prove challenging.

### Analysis frameworks and statistical tools

To be valuable in an applied context, neuromarkers need to provide information about a variable of interest. Such models necessitate knowledge of an outcome category or score corresponding to each dataset. In machine learning terms, this type of analysis is known as *supervised* learning. As previously noted, the metrics that we currently use to delineate and define mental disorders may not be ideal. A true redefenition of diagnostic categories and disease entities based on biological data, as advocated by the RDoC, requires a different approach. In machine learning, *unsupervised*, or *data mining* approaches work independently of outcome categories or dependent variables, attempting to cluster the data into coherent groups based on the information provided. In the case of psychiatric neuroimaging this takes the form of grouping participants into sub-categories based on their brain structure or function, independent of symptomatology. An example of this approach comes from the field of ADHD: Costa Dias et al. (58) examined resting state functional connectivity in a sample of 106 children, 43 of which were diagnosed with ADHD. This study identified three ADHD neurotypes (see Glossary) characterized by differences in functional network structure. It is feasible that the neurotypes identified in this study might represent distinct etiological pathways. Since the neurotype groups also differed with regard to impulsivity and activity level, the core deficits in these groups appear to be at least partially distinct, making it likely that treatment approaches may differ in terms of success between neurotypes. Clustering studies that have direct clinical utility link neurotypes to clinically relevant outcomes. An early series of studies combined cluster analysis with prediction of treatment outcome in a group of cocaine users (59, 60). These studies found that unsupervised clustering of resting-state electroencephalography data could group cocaine users into groups which differed in terms of the length of their stay in a treatment facility. The neurotypes discovered in this study have no ability to reveal etiological pathways, but they do provide some evidence of neurobiological characteristics associated with recovery, which is an equally important aspect of psychiatric neuropathology. In contrast, a more recent study linked treatment outcomes in depression to neurotypes (61). This study was able to identify clear differences in likelihood of certain symptoms presenting and overall disorder severity between neurotypes, thereby creating a link between the biological disorder subtypes and clinical presentation.

Given a known outcome variable, such as treatment success, neuroimaging research has typically employed group-difference analyses to identify factors associated with this outcome. Recognition of the limitations of this approach has led a large number of authors in psychology and neuroscience to emphasize the importance of moving away from explanatory and univariate analysis procedures and toward multivariate outcome prediction (21, 31, 34, 62). In the past decade the number of neuroimaging studies using multivariate methods has grown rapidly (34), and there is a strong recognition of the importance of this approach [(30, 62–64)]. The divergence of findings using classic univariate compared to multivariate methods is demonstrated by two recent meta-analyses summarizing neuroimaging studies of unipolar depression: There was a notable lack of significant differences in brain activity during emotional or cognitively challenging tasks associated with unipolar depression using traditional group comparison studies (65); However, a meta-analysis of studies using a multivariate approach to classify patients with major depressive disorder and healthy control subjects found an average classification accuracy of around 75% for functional MRI (66).

Supervised learning using multivariate models requires a particular set of statistical tools that departs strongly from the traditional group-difference approach. When using multivariate analysis methods, it is of great importance that the analysis protocol include some measures to prevent overfitting. The most fundamental of these is that a model must be tested on a previously unseen sample in order to obtain a realistic estimate of model accuracy. This step is crucial, as it is the most effective way to gauge how well a model will perform with other individuals from the same population. Using a separate dataset is the gold standard in terms of assessing external validity. However, a more easily accessible method is cross-validation (CV). One of the most frequently used methods is leave-one-out CV [LOOCV; e.g., (67–69)], or leave-k-out CV [e.g., (70)]. A somewhat less computationally expensive method is k-fold CV (e.g., (71)]. When using CV it is imperative to ensure that the observations used to validate the model (the test set) remain statistically pure and do not at any point overlap with the observations used to create the model [the training set; (72)]. Another tool that is important in quantifying in-sample generalizability is bootstrapping. Bootstrapping improves the stability of a model by randomly sampling the dataset with replacement multiple times in order to minimize the effect of outliers and estimate the true population mean (73). In particular, bootstrapping provides a measure of how reliable and consistent coefficient estimates or feature metrics are with datasets that have a low signal-to-noise ratio (see Glossary) and high multicollinearity. Bootstrap aggregation (bagging) has previously been used with large genetic datasets, and showed significant improvements over standard (non-bagged) methods in terms of model accuracy and stability (74). Both cross-validation and bootstrapping can be considered “resampling” procedures, and are standard tools used in Machine Learning.

Another important step which should be implemented when working with high-dimensional neuroimaging data is dimensionality reduction. Dimensionality reduction simply refers to the reduction of the number of variables that will be used to create a model. Dimensionality reduction approaches can be broadly categorized into “feature selection,” and “feature extraction” methods. Feature selection takes the existing input features and strategically removes those features that will, or are most likely to, contribute little to the accuracy of the model. Feature selection methods can be categorized into *filter* methods, *wrapper* methods, and *embedded* methods. These differ in terms of how the selection of features included in the regression model and model optimization (or learning) interact. Filter methods rank all brain inputs by factors such as their correlation with the target variable (i.e., prediction accuracy), and the most informative variables are selected. This is often used to initially reduce the size of datasets before other feature selection methods are used. Wrapper methods (75) use a learning machine (e.g., sequential search algorithms) to evaluate the quality of subsets of features [see (70)], thereby accounting for the importance of feature interaction effects. In contrast to filter and wrapper approaches, model building and feature selection cannot be separated in embedded methods. Some of the most common embedded feature selection algorithms are regularization methods, which penalize model complexity as a part of function optimization. Examples of these methods include Ridge, Lasso, and Elastic Net regularization (76). The Elastic Net has gained popularity among neuroimaging researchers in recent years, and has been successfully used in a number of large studies [e.g., (71, 77)]. While the method of feature selection does not seem to make a difference for large genetic datasets (78), embedded methods have been shown to be more effective than filter methods with certain neuroimaging classification problems (79). A more in-depth discussion of filter and wrapper methods can be found in Chandrashekar and Sahin (80), and Mwangi et al. (81) provide a review of feature selection techniques and their application to neuroimaging data.

In contrast to feature selection, feature extraction methods such as principal component analysis (PCA) and independent component analysis (ICA) are very familiar to neuroimaging researchers. Data scientists in other domains routinely use feature extraction techniques to map features onto higher-level summary variables to reduce the dimensionality of the dataset. Feature extraction always involves creating a new set of features from the original input variables, which normally makes the model difficult to interpret. It is therefore very complicated to evaluate whether a model is neurophysiologically plausible when feature extraction methods are used. While feature extraction methods often results in an improvement in model accuracy, they have largely been avoided by neuroimaging researchers when seeking to identify neuromarkers. However, there have been some advances that capitalize on the improvement in accuracy which can be gained from feature extraction methods, while also mapping results back onto the original feature space (82).

In addition to feature selection and feature extraction, it is also possible to manually create summary variables based on domain knowledge. This is referred to by Hahn et al. (13) as “feature engineering.” Feature engineering is a supervised form of feature extraction that capitalizes on the researchers' domain knowledge to create features that represent the underlying problem in a superior way. While this approach holds promise in that it makes it possible to integrate previous knowledge and theoretical understanding of a disorder directly into the model building process, we believe that some caution is warranted. In the same way that our current understanding of the neurobiological and psychological processes underlying mental disorders depends both on the state of the neuroscientific evidence and on the available theoretical frameworks, there is a danger that feature engineering may bias findings toward results that support a particular theoretical model of neurobiological processes. At the very least researchers should be aware of this caveat, and clearly communicate that their analysis framework is not purely data-driven, but incorporates at least some elements of theory-driven analysis (83).

Finally, despite efforts to guard against overfitting, there may nevertheless be a degree of unwarranted optimism in any model. Establishing whether a model produces results that are significantly better than chance is therefore not possible using traditional *p*-values. Rather, an empirical significance threshold should be established using a null model (i.e., a model against which the observed data can be compared to determine the likelihood that any observed effect could have occurred by chance). A commonly used approach to generating null model data is a simple randomization of the dependent variable across participants (random label permutation). Other approaches to constructing null models and null data include randomizing input data, and use of only nuisance covariates (23). The level of accuracy achieved by the analysis framework using this null data is compared to the accuracy of the model with real data, and this acts as a measure of the optimism inherent in the analysis framework.

### The neuromarker development pipeline

The developmental pipeline for neuromarkers in psychiatry should be very similar to the standard drug development pipeline. Woo et al. (34) and Moons et al. (19, 84) have laid out this developmental pipeline for biomarkers, making specific recommendations and providing a tangible way to evaluate how close to clinical applicability biomarkers are. The number of participants required increases the further along the road to clinical applicability a model is (34, 84). Initial exploratory studies typically have small sample sizes and modest resources, but the findings from these studies can be used to justify investing a higher amount of resources for further research and development (21, 34). At this stage it is advantageous to pursue many different avenues in terms of modalities and functional tasks in order to find the approach that best predicts the outcome. Generally, the most efficient approach to biomarker development will take into consideration what we already know at every stage of the development pipeline (19). In the initial stages of neuomarker research this may take the shape of selecting functional imaging tasks to use based on previous research. When analyzing the data, this may include the use of targeted feature engineering as suggested by Hahn et al. (13), taking into account the caveats of this approach. Woo et al. (34) estimated that around 450 models in the exploratory stage of development had been published in January 2016 relating to mental disorders (excluding substance use).

After the initial creation of a biomarker, the next step is the application of the model to an independent sample. This serves the purpose of initial generalizability testing. Woo and colleagues estimate that only around 40 neuromarkers have been validated using independent samples. Jollans and Whelan (62) provide a summary of some of these studies from the domains of major depressive disorder (85), psychosis (86–88), and dementia (89–91). Only two neuromarkers were identified by Woo et al. (34) that had also been validated using data from another data collection site. One of these is the SPARE-AD classifier (89), an overview of which is given below. Biomarker models should be treated as shareable research product, to be updated, validated, and amended by other research groups (13, 34, 84). Testing in other laboratories is an important measure of model performance because differences between a variety of cohorts from the same population on occasion have much larger effect sizes than differences between groups within the population [for example, typically developing and ASD individuals, (92)]. While unimodal models are easiest to test in other laboratories, generalization studies (see Glossary) should also examine what additional measures can enhance a model (84). Examples of such an expansion of an existing neuromarker are given in a study by Davatzikos et al. (93), who included additional predictors alongside the SPARE-AD value (described further below), and in a study by Drysdale et al. (61) who found that an index of depression neurotype used in conjunction with a connectivity index was most successful in predicting treatment response. Multiple unimodal models can effectively be integrated using strategies such as “voting,” “boosting,” or other ensemble methods [(13); see Glossary]. In fact, combining multiple modalities in a single model typically results in higher model accuracy. Multimodal models (see Glossary) are also preferable from a theoretical perspective when attempting to describe the neurobiology underlying a given outcome (21, 34, 63, 94).

An essential element of model validation is testing for construct validity. That is to say, a good neuromarker must actually measure the concept that it is assumed to measure. This seems straightforward, but in many cases the substantial phenotypic overlap between disorders may make it difficult to pinpoint what aspect of a disorder a biomarker is measuring, and in what context it will perform poorly. An example of this could be a classifier that supposedly separates control subjects from individuals with substance use disorder. It is conceivable that such a biomarker may in fact tap into externalizing symptoms common to ADHD and substance use disorder, or inadvertently identify individuals with ADHD symptoms, since they have higher substance use risk than those without ADHD symptoms (95). A biomarker assumed to measure a particular concept should therefore also be tested using populations which it should not have any relevance to, as well as populations to which it is assumed to generalize well. Ideally, biomarkers should be tested on very large, population-level samples that include a range of “confounds.”

Validating neuromarkers developed to differentiate among disorder subtypes is a specific challenge. For example, a study may identify a neurotype indicative of treatment outcome and provide a characterization of the neurotype based on symptomatology. However, given the difficulty (or indeed impossibility) of defining discrete disorder subtypes, indicators of neurotype could be integrated into models that predict other clinical outcomes such as treatment success [see (61)].

Finally, the ultimate test of the clinical utility of a biomarker should be large-scale randomized control trials, evaluating outcomes for patients who were assessed using traditional methods and patients who were assessed with the help of the biomarker (84). This step will serve as a measure of how much use of the biomarker actually contributes to patient care in an applied healthcare setting. At this point weighing up the cost and the benefits of the biomarker will determine whether it is suitable for integration into healthcare settings.

### Not so far off after all: a neuromarker for Alzheimer's disease

The psychiatric domain which has seen the largest amount of neuromarker research and for which some of the most promising neuromarkers have been developed is Alzheimer's disease (34). This is largely due to the freely available ADNI database. ADNI includes data from older adults who are cognitively normal (CN), diagnosed with mild cognitive impairment (MCI), or with Alzheimer's Disease (AD). This stratification of participants along what can be regarded as a continuum of cognitive impairment and dementia represents a more ecologically valid sampling scheme (i.e., better represents the population) than many psychiatric neuroimaging studies. The ADNI study collected longitudinal data from older adults, capturing cognitive decline and transition from CN to MCI and from MCI to AD. Due to the large sample size and range of impairment present in this sample, researchers were able to use subsets of participants to develop and validate a neuromarker. The SPARE-AD classifier was originally developed using 66 CN and 56 AD participants (89). The classifier generates a score which separated the AD and CN group in this sample with 94% accuracy. To validate the classifier, data from a group of 88 MCI participants were used. The classifier separated the MCI and CN group with 82% accuracy. Accuracy was 74% when separating the MCI and AD group. While confirming that the classifier has strong validity in detecting characteristics unique to AD, these results also show that the classifier detects factors associated with cognitive decline more generally. The next step in terms of model validation in this study was the classification of MCI patients according to their SPARE-AD score into a group likely to develop AD and a group likely not to develop AD. Based on participants' cognitive decline over the next 3 years the classification accuracy of MCI individuals (*n* = 38) was 87%. The classifier trained in this study was subsequently applied to a different sample of MCI patients from the ADNI cohort (93). This represents the first stage of generalization tests for this model. While the classification reached 90% sensitivity (*n* = 69 patients transitioned to dementia), only 37% of the MCI patients who remained stable within the timeframe of the study (*n* = 170) were correctly classified (56% classification accuracy). This result demonstrates the importance of generalization tests to obtain a realistic estimate of how well the neuromarker performs.

SPARE-AD has also been applied to data from the Baltimore Longitudinal Study of Aging (BLSA). Validating the classifier on a sample drawn from a different geographical location is an important step in determining external validity. Davatzikos et al. (96) used data from 109 CN participants who did not transition to MCI over a 14 year period and data from 15 individuals who transitioned from CN to MCI. They were able to predict whether or not individuals would transition to MCI based on the rate of change of their SPARE-AD score (AROC = 0.89; see Glossary). Davatzikos et al. (93) integrated the SPARE-AD classifier and a cerebrospinal fluid marker, which improved classification accuracy from 56 to 62% (84% sensitivity, 51.2% specificity). Further evidence from a study comparing various AD classifiers found that SPARE-AD in combination with a measure of cognitive performance and genotype information provided the highest classification accuracy (97). Integrating multiple classifiers to improve accuracy is another important step toward developing a clinically useful biomarker.

Both studies using the ADNI database used samples including more than 200 participants, and in total the SPARE-AD classifier has been tested on more than 550 individuals between the studies described here. Using larger than average samples from multiple data collection sites made it possible to develop a neuromarker of cognitive decline in older adults, which has been shown to reliably identify individuals at risk for future cognitive decline. While the rate of false positive identification was shown to be quite high for this classifier, it is a validated tool that could feasibly be applied in clinical contexts to make a reasonable estimate of risk of cognitive decline in older adults. Based on the encouraging results obtained in these studies, SPARE-AD should continue to be tested across laboratories and scanners to reach the final phase of the model development pipeline: population-level generalization (34).

#### Summary

In this section we discussed the tools necessary to develop neuromarkers for mental disorders. Studies that seek to identify or test neuromarkers must take into consideration that the population from which their sample is drawn will also be the only population to which findings can be expected to generalize. Furthermore, it is imperative that researchers make use of freely available large datasets or collect data from large samples. Studies that include a large number of participants with a wide range of symptoms, and collect not only imaging data but also genetics, demographic data, and so on have the potential to produce the most clinically useful findings. Whether researchers use supervised or unsupervised analysis methods will depend on the question which they seek to answer. Supervised learning is preferable when a definitive outcome (such as relapse or disease course) is known, whereas unsupervised learning may be more beneficial when the outcome is not so clear (such as subtypes of diagnostic categories). For supervised learning approaches, rigorous generalization testing through resampling methods is crucial. Reducing the number of features included in the model through feature selection can help to prevent overfitting. Other dimensionality reduction strategies are available, but researchers should be aware of the practical and theoretical implications of choosing them. Significance should be established using null models. To reach clinical applicability, neuromarkers must undergo extensive generalization testing in other laboratories, with other populations, in combination with other biomarkers, and finally in randomized controlled trials. Due to many researchers' reluctance to use neuromarkers established in other research groups, most neuromarkers have not undergone generalization tests using other samples. An exception to this is the SPARE-AD classifier of Alzheimer's disease.

## What to watch out for with neuromarkers—practical and ethical considerations

With the goal of using reliable neuromarkers in psychiatry come a number of ethical considerations that should be kept in mind by researchers and clinicians who are working toward integrating neuromarkers into clinical assessments. The most immediately relevant consideration concerning research in this field is resource allocation. Resource allocation for neuromarkers research should be based on the effectiveness of treatment using current standard tools. That is to say, groups that are not well-served by current diagnostic, prognostic, or treatment approaches should be the primary target of psychiatric neuromarker research (33). Furthermore, rather than continuing to develop new potential neuromarkers in domains which have already identified potential biological models, research efforts should go toward validating and updating or expanding the most promising existing models. Adopting a component process approach may contribute to the re-use of models across laboratories.

With increased focus on brain-based measures of psychiatric conditions also comes the challenge of maintaining a holistic view of psychiatric disorders as being caused by multiple diverse etiological factors (98). While there may be a temptation to prioritize brain-based methods of explanation and treatment, there is an important balance to be struck between undervaluing advances based on neuroimaging, and scientific reductionism which discounts treatments and modes of interpretation based on the mind in favor of the brain. To what extent psychiatric science based on domains other than biology will continue to be useful will remain to be seen. As noted by Kendler [(98), p. 385], “having a realistic view of the causal landscapes of psychiatric disorders can only help.”

Another caveat to consider is that predictions are not deterministic. In the area of healthcare this is of particular importance, as risk assessments and prognoses based on biological measures may well go on to inform the cost of health insurance. On the other hand, neuromarkers indicating the most promising treatment pathway should be seen as objective support for the clinician's choice of treatment, and should therefore factor into the level of contribution from health insurance providers to the cost of treatment. Finding satisfactory middle ground will be complex in this area, and will require that both researchers and clinicians engaged in neuromarker development are aware of the possible implications of their work for the patients they aim to benefit.

### Summary

Researchers and clinicians should remain aware that moving from the phenomenological framework in psychiatry to a more biologically defined approach has implications for how mental disorders will be approached by stakeholders in the healthcare industry. Furthermore, the goal of neuromarker research should be to improve upon current diagnostic and prognostic capabilities as much as possible, which means that resources should be allocated where neuromarkers are most promising and where they are already in development.

## Summary and conclusion

In this review we have discussed why neuroimaging research has not had a substantial influence on psychiatric practice to date, and what is required to work toward identifying truly useful neuromarkers—neuroimaging biomarkers—of mental disorders. We provided an outline of the tools that are typically used in neuropsychiatric research investigating mental disorders, and argued that research should be focused toward developing neuromarkers for use in applied psychiatric settings. We presented problems inherent in the traditional group comparison approach used in psychiatric neuroimaging research, and suggested that broader, more population-based study designs will be likely to have greater clinical utility. To move toward an approach that places higher emphasis on individual differences, it is necessary to work with large samples. It is also advantageous not to rely on current diagnostic criteria for mental disorders when working toward neuroimaging biomarkers, as these may not correspond to biological subtypes of mental disorders. Identifying neuroimaging biomarkers will only be possible if researchers adopt multivariate regression-type approaches, and machine learning analysis frameworks. The generalizability of findings should be given highest priority. Therefore, resampling methods such as cross-validation must be used at a minimum, with external validation as the “gold standard.” Furthermore, neuroimaging data calls for the use of dimensionality reduction approaches such as feature selection. In order to determine the validity of a model it is necessary to be able to examine whether it is neurophysiologically plausible, making interpretability an important concern when constructing models. Before they can be implemented in healthcare settings, neuromarkers must be tested using large diverse samples, across a range of geographical locations and research groups.

We believe that a fundamental shift in how research in neuropsychiatry is conducted is necessary in order to produce viable neuromarkers. First, it is essential that researchers not rely solely on current diagnostic criteria when developing neuromarkers. These criteria are unreliable, based only on behavioral and self-reported symptoms, and they misrepresent the etiology and underlying neuropathology of many mental disorders. While we believe that it would be advantageous for psychiatric nosology to be redefined based on neuroscientific evidence, the most efficient approach to neuromarker development will likely include a move toward component-process based models, and a focus on individual differences across populations. This will require a significant shift in how researchers approach study design and analysis. The focus of neuromarker research must be on external validity, on the accurate representation of a model's performance, and on the attainment of clinically useful neuromarkers. This will require the cooperation of publishers and funding bodies to gather and distribute knowledge about what approaches appear promising and what approaches do not, and to prompt and support replication and generalizability studies. Specifically, a population neuroscience approach including samples with a wide range of symptoms, and data from multiple neuroimaging and non-imaging modalities will be necessary to create far-reaching and effective neuromarkers.

In conclusion, we believe that the field of neuromarker research offers exciting prospects for the future of psychiatric healthcare. As was shown with the example of the SPARE-AD classifier, it is possible to create well-validated neuroimaging biomarkers that augment existing prognostic capabilities. Biological models of mental disorders have the potential to identify individuals at risk of developing psychiatric illness, or those in the early stages of neurodevelopmental and neurodegenerative disorders. This knowledge would be a valuable tool to identify those individuals who would most benefit from early interventions or from periodic monitoring. Psychiatry is currently decades behind most other areas of medicine when it comes to the use of objective assessments of disorder status or risk. However, we believe that the integration of big data and machine learning, which is already taking place in neuroscience and psychology, will allow us to not only improve healthcare through the integration of neuromarkers, but to also gain a much better understanding of the neurobiology associated with the development of, and recovery from, mental disorders.

## Author contributions

All authors listed have made a substantial, direct and intellectual contribution to the work, and approved it for publication.

### Conflict of interest statement

The authors declare that the research was conducted in the absence of any commercial or financial relationships that could be construed as a potential conflict of interest.

## Glossary

**Neuromarker**: A neuromarker is a biomarker based on neuroscientific data, such as neuroimaging. Neuromarkers are biological indicators of the presence or progression of a disease or condition. They are generally statistical models that provide an objective estimate of how likely it is that a given condition is present. Neuromarkers can consist of a single variable, or be complex multivariate models.

Brain imaging terms:

**Voxel**: A voxel can be thought of as a three-dimensional pixel. Voxels are the smallest units in three-dimensional brain images obtained using MRI.

**Region of interest (ROI):** ROI is a term used in neuroimaging to describe data which contain information about a specific area of the MRI image. ROIs will often correspond to predefined regions within the brain, such as the amygdala or the hippocampus. Data within an ROI are typically averaged to include in statistical tests.

**Signal to noise ratio (SNR):** In imaging, SNR refers to the ratio of signal within the data to the background “noise.” In brain imaging terms this represents the strength of the signal coming from the brain itself, such as from the BOLD signal, compared to the (random) background noise which is of no interest. SNR is normally low in neuroimaging.

Psychological terms:

**Symptomatology**: For mental disorders, symptomatology refers to observable and self-reported symptoms which an individual experiences. This may include physiological and psychological symptoms.

**Phenomenology**: In psychology, phenomenology refers to the description of an individual's experience, and is dissociated from objective reality.

**Neurotype:** A neurotype, or biological subtype of a disorder or condition is a subset of the population that shows particular characteristics of brain structure or function.

Statistical and machine learning terms:

**Inferential statistics**: The *t*-test is a test of the statistical hypothesis that two samples are drawn from the same population. The underlying assumption of the *t*-test is that data from the same population would follow a normal distribution. *T*-tests are often used to test whether there are statistically significant differences between two groups., Generally, an Analysis of variance (ANOVA) is the extension of the *t*-test to multiple groups. ANOVAs test for differences in group means.

**Model (Statistical model)**: A statistical model refers to the formal description of the generation of data. Statistical models can be thought of as mathematical representations of theories. Statistical models usually describe the relationship between one or more independent variables (such as neuroimaging data), and some dependent variable of interest (such as symptomatology). The multivariate models referred to throughout the text typically have multiple input variables that are weighted depending on how strongly they contribute to the description of the dependent variable. The weighted input variables are then combined in a mathematical equation that results in an estimate of the outcome variable.

**Unimodal and multimodal models**: Unimodal models include only data from one domain, such as a single type of neuroimaging data. Multimodal models include data from more than one modality.

**Ensemble methods**: Ensemble methods make it possible to use multiple statistical models to create a summary model. Examples of this approach are “voting,” and “boosting.” Ensemble methods often combine results from multiple models into a new model, weighting inputs to create a superior estimate than would have been achievable using each model on its own.

**False positive:** False positive results are findings which indicate that something is true, when it is in fact not true., False positives are often used to describe the results of classification studies where a member of the negative class (typically control participants) may be erroneously classified as a member of the positive class (typically patients).

**Sensitivity and Specificity**: Sensitivity refers to the number of cases from the positive class (typically patients) that were correctly identified by the model, and specificity refers to the number of cases from the negative class (typically control participants) that were correctly identified.

**Area under the curve of the receiver operating characteristic curve (AROC):** The AROC refers to the integral of the receiver operating characteristic curve (ROC). The AROC is a frequently used metric of model fit for classification models and logistic regression. The ROC curve tracks the rate of true and false positive classification of the model. The true and false positive values are on a continuum where the extremes are the instances when all cases are classified as elements of one class. Higher AROC values denote better model fit, and a higher rate of true than false positive classification. The maximum AROC value is 1, with .5 representing chance performance.

**Generalization study/test**: A generalization study uses a sample that is independent of the dataset that was used to create a model. The generalization study is used to test how well a model performs when it is applied to a different sample.

**Clustering**: Clustering or cluster analysis is a data-driven approach which groups datapoints such that datapoints within the same group (cluster) are more similar to each other than to datapoints outside the group.
